# The Skin Bacterium *Propionibacterium acnes* Employs Two Variants of Hyaluronate Lyase with Distinct Properties

**DOI:** 10.3390/microorganisms5030057

**Published:** 2017-09-12

**Authors:** Seven Nazipi, Kristian Stødkilde, Carsten Scavenius, Holger Brüggemann

**Affiliations:** 1 Department of Biomedicine, Aarhus University, 8000 Aarhus C, Denmark; seven90@live.dk (S.N.); kst@biomed.au.dk (K.S.); 2 Department of Molecular Biology and Genetics, Aarhus University, 8000 Aarhus C, Denmark; csss@mbg.au.dk

**Keywords:** *Propionibacterium acnes*, hyaluronate lyase, hyaluronic acid, glycosaminoglycan, extracellular matrix, skin microbiota

## Abstract

Hyaluronic acid (HA) and other glycosaminoglycans are extracellular matrix components in the human epidermis and dermis. One of the most prevalent skin microorganisms, *Propionibacterium acnes*, possesses HA-degrading activity, possibly conferred by the enzyme hyaluronate lyase (HYL). In this study, we identified the HYL of *P. acnes* and investigated the genotypic and phenotypic characteristics. Investigations include the generation of a *P. acnes* *hyl* knockout mutant and HYL activity assays to determine the substrate range and formed products. We found that *P. acnes* employs two distinct variants of HYL. One variant, HYL-IB/II, is highly active, resulting in complete HA degradation; it is present in strains of the phylotypes IB and II. The other variant, HYL-IA, has low activity, resulting in incomplete HA degradation; it is present in type IA strains. Our findings could explain some of the observed differences between *P. acnes* phylotype IA and IB/II strains. Whereas type IA strains are primarily found on the skin surface and associated with acne vulgaris, type IB/II strains are more often associated with soft and deep tissue infections, which would require elaborate tissue invasion strategies, possibly accomplished by a highly active HYL-IB/II.

## 1. Introduction

*Propionibacterium acnes* is a Gram-positive anaerobic but aerotolerant bacterium present in sebaceous follicle-rich areas of human skin, such as the face, upper chest, and back. The bacterium is a major commensal of the human skin, accounting for a large proportion of the total human skin microbiota [1]. Despite its low virulence, it is commonly known to play a role in the pathogenesis of the skin disorder acne vulgaris [2,3]. The bacterium has also been associated with opportunistic infections such as prosthetic joint infection, sarcoidosis, endocarditis, and several other diseases [4,5]. Based on several phylotyping schemes and complete genome sequencing, the population of *P. acnes* consists of several phylogenetic subtypes commonly designated IA1, IA2, IB, IC, II and III [6,7,8,9,10,11]. It was recently proposed to reclassify type I strains as *P. acnes* subsp. *acnes*, type II as *P. acnes* subsp. *defendens* and type III as *P. acnes* subsp. *elongatum* [12,13]. Moreover, it was proposed to rename *P. acnes* to *Cutibacterium acnes* [14].

Phylotype IA strains are predominate on normal skin, and certain subtypes of type IA appear to be predominately associated with acne-affected skin [7,9,15]. In contrast, phylotype IB, II, and III strains are rarer on facial and upper back skin and not associated with acne-affected skin. However, type IB and type II strains have been associated with various soft and deep tissue infections such as blood and medical device-related infections [7,10,16]. Type II strains were also frequently isolated from cancerous prostate tissues [17,18]. Type III strains are relatively rare on the skin of the face and upper back but are more often found on the lower back; they have recently been associated with the skin disorder progressive macular hypomelanosis [19,20].

In a recent study, genomic differences between the different *P. acnes* phylotypes were mapped and catalogued [21]. One genomic difference between type IA and type IB/II strains concerned the gene *hyl* that encodes a hyaluronic acid lyase/hyaluronidase (HYL). HYLs are ubiquitous enzymes that function primarily in the degradation of hyaluronic acid (HA). HA is a non-sulfated glycosaminoglycan (GAG) of high molecular weight; it is composed of repeated units of β-1,4-glucunoric acid and 1,3-*N*-acetylglucosamine disaccharides, connected through a β-linkage [22]. The molecule is an abundant extracellular polysaccharide in the epidermis and the dermis; up to 56% of the total body HA resides in the skin [22,23,24]. Both keratinocytes in the epidermis and fibroblasts in the dermis produce HA. Other glycosaminoglycans (GAGs) like chondroitin 4-sulfate (CSA), dermatan sulfate (CSB) and chondroitin 6-sulfate (CSC) are also degraded by both vertebrate and bacterial HYLs, but at a considerable slower rates [25,26].

Bacterial HYLs are found in many Gram-positive microorganisms including species of *Clostridium*, *Propionibacterium*, *Streptococcus*, *Staphylococcus* and *Streptomyces* [25]. Two groups of bacterial HYLs exist based on degradation patterns and substrate specificities. Group I enzymes degrade HA completely into disaccharide units, following a processive degradation mechanism. Group II HYLs degrade HA by means of a non-processive degradation mechanism into a mixture of unsaturated oligosaccharides and are specific for HA [27,28,29]. HYLs are often described as virulence factors that facilitate the spread of bacteria in tissue [30,31]. In addition, HA fragments produced by bacterial HYL activity can be used as nutrients [31]. Such HA fragments also have the ability to interact with host cells and modulate for instance host immune defenses during bacterial colonization [32]. These effects depend mainly on the size of the produced HA fragments [24,32,33,34].

HYL activity of *P. acnes* was first discovered in the late 1960s, and since then only a few studies have focused on the characterization of HYL in *P. acnes* [35,36,37,38]. Determination of HYL activity has been used as a diagnostic tool to identify *P. acnes* in clinical samples [39]. The HYL of *P. acnes* has been classified as true bacterial HYL based on sequence homology with other bacterial HYLs as well as enzymatic properties [36,37,38].

In this study we identified, investigated and compared the HYL activities of different *P. acnes* phylotypes. Results revealed striking geno- and phenotypical differences between the HYLs of the *P. acnes* phylotypes IA and IB/II, respectively. The study could help to explain phylotype-specific properties of *P. acnes* regarding its tissue invasion potential.

## 2. Materials and Methods

### 2.1. Bacterial Strains

*P. acnes* wild-type strains used in this study are listed in Table S1. Their MLST and SLST sequence types were previously determined [21,40]; the strains represent the *P. acnes* phylotypes IA, IB, II and III. Reinforced Clostridial Agar (RCA) was used as the solid growth medium. Strains were grown on RCA plates under anaerobic conditions in a 37 °C incubator. Brain heart infusion (BHI) medium was used as broth medium for liquid culture growth.

### 2.2. DNA Isolation and PCR Assay

DNA was isolated by the MasterPure^TM^ Gram Positive DNA Purification Kit (Epicentre, Madison, WI, USA). Purified genomic DNA was eluted in 50 µL of TE buffer and stored at −20 °C. The DNA concentration and purity was measured by Nanodrop. To differentiate the two *hyl* variants of type IA and type IB/II strains, respectively, a PCR assay was established; the *hyl* variant-specific primers are listed in Table S2. These primers were designed to amplify 500 bp fragments of the two *hyl* variants. PCR reactions were performed in 0.5 mL PCR tubes, which contained 10 μL of 5′-PRIME Hotmastermix (5 PRIME), 2 μL of primer mix, 1 μL genomic DNA, and 12 μL of PCR grade H_2_O. Amplification was achieved with an initial cycle of 96 °C followed by 35 cycles of 35 s of denaturation at 96 °C, 40 s of annealing at 55 °C and 40 s of extension at 72 °C. A final extension step was carried out for 7 min at 72 °C.

### 2.3. Phylogeny and Genome Comparison

Nucleotide and amino acid sequences of *hyl*/HYL of sequenced *P. acnes* genomes were extracted from GenBank. PPA0380 of strain KPA171202 served as *hyl* reference sequence [41]. Sequence alignment and phylogeny were done in MEGA 6.06, using MUSCLE and minimum-evolution algorithms, respectively. To compare the genomic context of the *hyl* gene, we used Sybil [42]. The Sybil database for *P. acnes* was established and described previously [21] and is accessible here: http://sybil-clovr.igs.umaryland.edu/sybil/ChristianScholz_Pacnes_sybil.

### 2.4. RNA Preparation and RNA Sequencing

Total RNA from *P. acnes* (KPA171202 (type IB), and 12.1.L1 (type IA)) grown under anaerobic conditions to the exponential and stationary growth phase in BHI medium at 37 °C was prepared as described previously [43]. The cDNA libraries were constructed by Vertis Biotechnology AG, Freising, Germany, as previously described [44,45]. Sequencing was carried out with a HiSeq 2000 instrument (Illumina, San Diego, CA, USA). Detailed descriptions of procedures used for quality control, read mapping, expression analyses and data normalization have been published previously [44]. For graph visualization the Integrative Genome Browser (IGB v8.5.4) was used [46].

### 2.5. Mutagenesis of P. acnes

Mutagenesis of *P. acnes* was done as described previously [43,47]. *P. acnes* strain KPA171202 was used as the wild-type strain. To amplify the 500 bp regions up- and downstream of the gene PPA0380 (*hyl*) the primer combinations PPA0380_1/PPA0380_2 and PPA0380_3/PPA0380_4, respectively, were used (Table S2). As selection marker, the erythromycin-resistance cassette *ermE* of *Saccharopolyspora erythraea* (DSM no. 40517) was used. The KPA171202 wild-type strain is susceptible to erythromycin. A protocol for preparing competent cells and electroporation settings were described previously [47]. After electroporation and one-day recovery, cells were plated out on agar plates containing 10 µg/mL erythromycin. Only few clones were obtained and tested by PCR to validate the correct knock-out of PPA0380. In addition, the Δ*hyl* mutant strain used in this study was sequenced to confirm that the *ermE* resistance cassette was inserted in the right location. 

### 2.6. Precipitation of Secreted P. acnes Proteins and Detection of HYL by Mass Spectrometry

*P. acnes* strains were grown in BHI broth to the stationary growth phase. Culture supernatants were harvested and sterile filtered, and proteins were precipitated using trichloroacetic acid (TCA) as described previously [48]. In brief, the supernatant filtrate was mixed with TCA to a final concentration of 10% TCA and incubated overnight at 4 °C on a tube rotator. The mixture was centrifuged for 20 min (20,000× *g* and 4 °C) and the resulting pellet was resuspended in 1 mL of ice-cold acetone, transferred to Eppendorf tubes and submerged into an ultrasonic bath for 10 min. The resuspended pellet was washed twice with acetone and the resulting pellet was air dried and stored at −80 °C. The pellets were dissolved in Laemmli buffer (20 % glycerol, 0.01% bromphenol blue, 2% 2-mercaptoethanol, 4% SDS, 125 mM Tris-HCl, pH 8) and boiled for 5 min before analyzed by SDS-PAGE (25 mA, 80 V). SDS PAGE was carried out with 12% acrylamide concentration of gels. Gels were stained with Coomassie Blue.

Protein bands in the MW range 80–90 kDa were excised for in-gel tryptic digestion. Nano-electrospray ionization MS/MS (nanoESI-MS/MS) analyses were performed on an EASY-nLC II system (Thermo-Fisher Scientific, Waltham, MA, USA) connected to a TripleTOF 5600+ mass spectrometer (AB SCIEX, Framingham, MA, USA) operated under Analyst TF 1.6.1 control. The trypsin-digested samples were suspended in 0.1% formic acid, injected, trapped and desalted on a precolumn. The peptides were eluted and separated on a 15 cm analytical column (75 μm i.d.), pulled in-house (P2000 laser puller; Sutter Instrument, Novato, CA, USA). Trap and analytical column were packed with ReproSil-Pur C18-AQ 3 μm resin (Dr. Maisch GmbH, Ammerbuch, Germany). Peptides were eluted from the analytical column at a flow rate of 250 nL/min using a 30-min gradient from 5% to 35% of solution B (0.1% formic acid, 100% acetonitrile). The collected MS files were converted to Mascot generic format (MGF) using the AB SCIEX MS Data Converter beta 1.1 (AB SCIEX, Framingham, MA, USA) and the “protein pilot MGF” parameters. The generated peak lists were searched using an in-house Mascot search engine (Matrix Science, Boston, MA, USA) against all *P. acnes* proteins in the UniProt database. Search parameters were allowing one missed trypsin cleavage site and propionamide as a fixed modification with peptide tolerance and MS/MS tolerance set to 10 ppm and 0.2 Da, respectively.

### 2.7. Hyaluronic Acid-Containing Plate Assay 

HA plates were prepared according to the method of Smith and Willet [35] with some modifications. BHI medium was mixed with 2% (wt/vol) of Noble agar (Difco, Thermo-Fisher Scientific, Waltham, MA, USA) and autoclaved. Two hundred milliliters of a 0.2% stock solution of HA sodium salt (Carbosynth, Compton, UK) was added to the cooled media together with 200 mL of a 5% (wt/vol) stock solution of bovine serum albumin (BSA) under constant stirring. The media was poured on plates and stored at 4 °C. Both HA and BSA were dissolved in water, sterile-filtered, and added to the agar medium. Colonies of *P. acnes* (harvested from RCA plates after 72 h of anaerobic incubation) were point-inoculated onto the surface of HA plates. For testing of *P. acnes* culture supernatants for HYL activity, supernatants were harvested from broth cultures (grown in BHI medium to stationary growth phase under anaerobic conditions) by centrifugation (3000 rpm, 30 min, 4 °C) and sterile filtration. 10 μL of the supernatants were spotted onto HA-containing agar plates. All plates were incubated for 24 h under anaerobic condition at 37 °C and flushed with 2N acetic acid for at least 15 min the following day. Clear zones around colonies indicate HA degradation, since degraded HA does not precipitate under acidic conditions. Recombinant HYL from *Streptococcus pyogenes* (Sigma-Aldrich, St. Louis, MO, USA) was used as a positive control for HA degradation.

### 2.8. Chondroitin Sulfate-Containing Plate Assay 

Chondroitin sulfate A (CSA), chondroitin sulfate B (CSB), and chondroitin sulfate C (CSC) plates were prepared according to Smith and Willet [35]. Briefly, CSA (Sigma-Aldrich, St. Louis, MO, USA), CSB (Sigma-Aldrich, St. Louis, MO, USA), and CSC (Carbosynth, Compton, UK) were dissolved in sterile water to obtain a 4 mg/mL stock solution. Preheated and sterile-filtered chondroitin sulfate solutions and 5% BSA were added to 1% BHI medium to obtain final concentrations of 400 μg/mL CSA, CSB or CSC and 1% BSA. Colonies of *P. acnes* (harvested from RCA plates after 72 h of incubation) were point-inoculated onto the surface of the plates and incubated anaerobically for either 24 h or 96 h. 2N acetic acid was poured on the plates after incubation and left to react for at least 15 min.

### 2.9. Hyaluronate Lyase Turbidimetric Assay

An additional assay was performed for a quantitative measurement of HYL production of *P. acnes*. The method was modified from the protocol of Tolksdorf and McCready [49]. *P. acnes* bacterial cultures with same start optical densities (OD_600_ = 0.05) were grown in 40 mL of BHI media for 24, 48, 72, and 144 h, respectively. After each time point, the OD_600nm_ was measured. Culture supernatants were harvested and sterile-filtered. 50 µL (of HYL-containing) supernatants from each time point was combined with 50 μL of 0.4% HA in a 96-well plate, and was allowed to react for 10 min under agitation at 37 °C. Each sample was done in triplicates and the experiment was repeated twice. After incubation, the samples were treated with 150 µL of BSA reagent (2.5 g of BSA dissolved in 250 mL of 0.5 M sodium acetate buffer, pH 4.2) for 10 min at room temperature. A multiscanner ELISA reader, adjusted to an optical density of 540 nm was used to determine the turbidity.

### 2.10. Detection of Hyaluronic Acid Fragments

The method to detect HA fragments after HYL incubation of HA was described previously [50,51]. One percent HA and (HYL-containing) culture supernatants were mixed (2:1) in Eppendorf tubes for 10, 20 and 30 min at 37 °C. The samples were subsequently treated with pronase from *Streptomyces griseus* (Roche, Basel, Switzerland) to terminate HYL activity. The HA fragments were visualized by loading the samples onto a 1% TopVision low-melting point agarose gel with an electroendosmosis of 0.11 (Thermo-Fisher Scientific, Waltham, MA, USA). For loading, 5 μL of sample was mixed with 3 µL loading buffer and 10 µL of PCR water. The content was loaded on the gel, which ran at 18 V for 1 h and then 35 V for 4 h. The gel was stained with a solution of 0.005% Stains-all dye (Sigma-Aldrich, St. Louis, MO, USA) dissolved in 50% ethanol. Stains-all dye is a cationic dye that binds to negatively charged HA fragments. The staining procedure was done in dark conditions at room temperature under agitation. The gel was de-stained with a 10% ethanol under same conditions as the staining procedure, and repeated at least three times. Further de-staining was accomplished by leaving the gel for 10 min in well-lit conditions.

## 3. Results

### 3.1. Genotypic Characterization of P. acnes Hyaluronate Lyase

**Figure 1:**
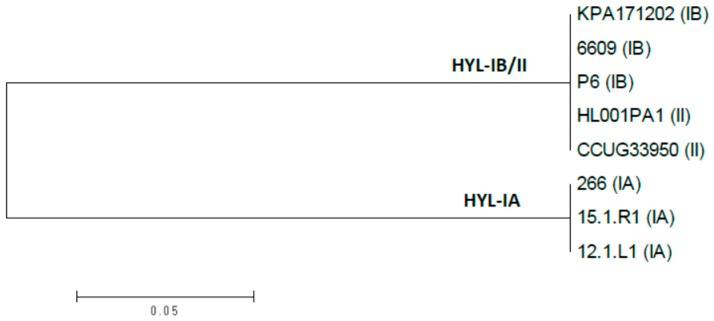
Phylogenetic tree of HYL homologs of *P. acnes* type IA, type IB, and type II strains. The phylogenetic tree is based on amino acid sequences HYLs from type IA, IB, and II strains. HYL sequences are separated into two phylogenetically distinct clades, HYL-IB/II and HYL-IA. All investigated type IA strains possess a HYL-IA variant, and all investigated type IB and II strains possess a HYL-IB/II variant. Type IC strains (not included) harbor a HYL-IA variant. The tree was constructed as an unrooted minimum-evolution tree in MEGA v. 6.06.

The HYL-encoding gene *hyl* has the gene locus tag PPA0380 in the reference genome of the type IB *P. acnes* strain KPA171202 [41]. It encodes an 813 amino acid protein of 88 kDa, harboring an N-terminal signal peptide for its secretion. A BLASTP search of PPA380 in all available *P. acnes* genomes revealed that PPA0380 has identical/highly similar homologs (>98% identify) in all type IB and type II strains; this HYL variant is hereafter named HYL-IB/II. In type IA strains and also in type IC strains, PPA0380 has a homolog with a considerably lower similarity, in average 74% identity on nucleotide as well as on protein level; this variant is hereafter named HYL-IA (Figure 1 and Figure S1). HYL-IA is 826 amino aacids large and has also an N-terminal signal peptide. Thus, on the basis of the HYL variant, the *P. acnes* population can be divided into two groups: comprising type IB/II and type IA/IC strains. No PPA0380 homolog could be found in type III strains.

**Figure 2:**
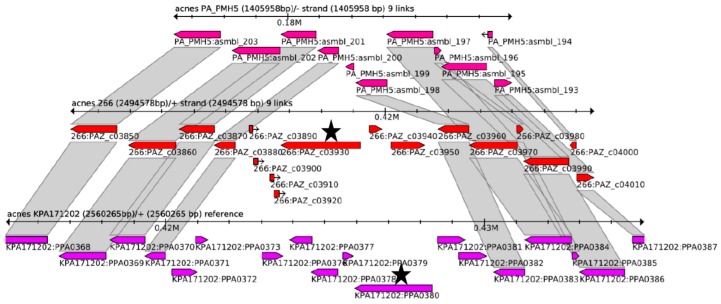
Genome context comparison of the region encoding HYL in *P. acnes*. The genomic region around *hyl* from strains PMH5 (type III), 266 (type IA) and KPA171202 (type IB) was compared using Sybil. The grey connecting blocks indicate amino acid sequences that share over 75% identity. The respective *hyl* genes are highlighted (black star). Figure S2 shows an extended comparison comprising more *P. acnes* genomes.

To analyse the genomic context of *hyl*, the Sybil comparison tool was used [42]. This analysis showed that the *hyl* gene is part of an island-like region that is inserted into the core genome in type IA, IB and II strains, but not in type III strains (Figure 2 and Figure S2). The island is 6.3 kb and 10 kb in size in the genomes of strain 266 (IA) and KPA171202 (IB), respectively. Interestingly, the genomic context of *hyl* in type IB/II and type IA strains differs, respectively, as seen by the absence of homology of the genes in the close vicinity up- and downstream of the *hyl* gene. This indicates that the *hyl*-containing island has been independently acquired in type IA and type IB/II strains, respectively. A *hyl* variant-specific PCR assay was successfully established in order to distinguish HYL-IA and HYL-IB/II in *P. acnes* strains (Figure S3). Taken together, the results show that two phylotype-specific variants of HYL exist in the population of *P. acnes*. One variant is found in phylotype IA/IC strains, while the other variant of HYL is found in phylotype IB and II strains, respectively.

### 3.2. Transcriptional Activity of Hyl in Type IA and IB Strains and Hyaluronate Lyase Production in Culture Supernatants 

**Figure 3:**
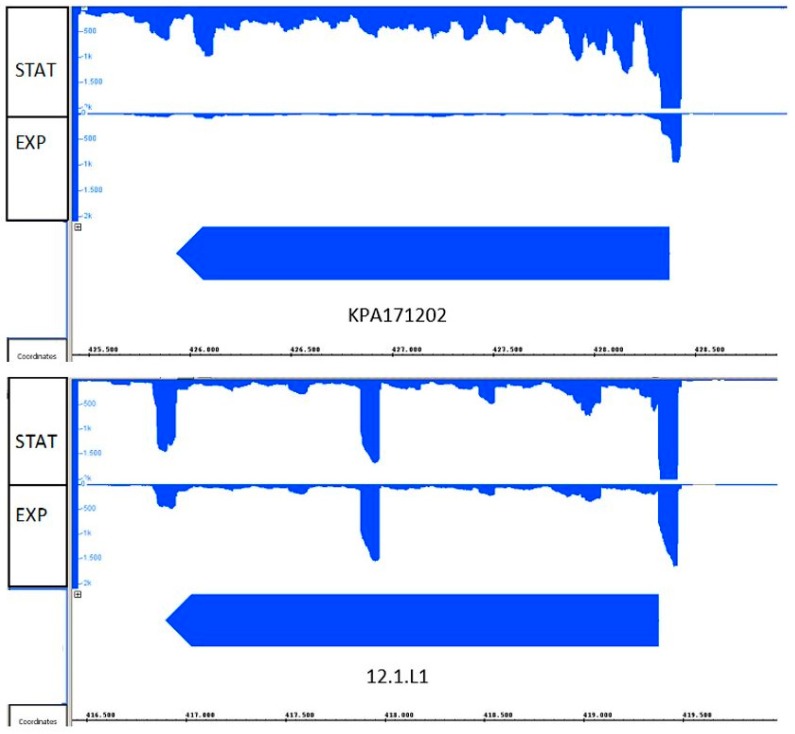
Transcriptional activity of *hyl* in *P. acnes* strains 12.1.L1 (type IA) and KPA171202 (type IB). RNA-sequencing was applied to record the expression of *hyl* in *P. acnes* strain KPA171202 (type IB, **top**) and 12.1.L1 (type IA, **bottom**). Transcription was recorded in bacteria grown to exponential (EXP) and stationary (STAT) growth phases. Both strains expressed *hyl*; strain KPA171202 exhibited stronger expression in the stationary growth phase.

To obtain additional insight in the two HYL variants, we determined the transcriptional activity of *hyl* in the strains KPA121702 (type IB) and 12.1.L1 (type IA). The strains were grown under identical conditions (BHI broth, anaerobic conditions) to exponential phase and stationary growth phases and RNA was extracted, sequenced with Illumina technology, and sequence reads were mapped to the respective reference genomes. The transcriptome data revealed that the *hyl* gene was actively transcribed in both of the investigated strains. The expression of HYL-IB/II (strain KPA171202) was growth phase-dependent with lower transcription during the exponential phase, followed by an increased transcription in the stationary phase (Figure 3). In contrast, HYL-IA (strain 12.1.L1) appeared to be equally transcribed in both growth phases. Deeper analysis of the transcriptome data exposed the transcriptional start sites as well as the putative -10 and -35 regions of the *hyl* promoters (Figure S4). The *hyl* gene of the two strains had different promoter regions and 5′-untranslated regions (5′-UTR); the 5′-UTR was longer in strain 12.1.L1 compared to KPA121702. Taken together, these findings showed that *hyl* is transcribed in the tested type IA and IB strains, albeit differences were identified.

**Figure 4:**
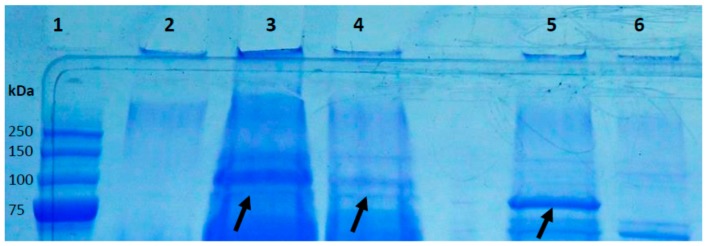
Hyaluronate lyase is a secreted protein of *P. acnes*. Secreted proteins of *P. acnes* grown to stationary growth phase, harvested from culture supernatants and separated by SDS-PAGE. Shown are secreted proteins above 75 kDa. All visible bands were excised and analyzed by MS. The bands that have been identified as HYL are highlighted with an arrow. Lanes: 1, MW marker; 2, control (BHI medium); 3, strain 266 (IA); 4, strain 12.1.L1 (IA); 5, strain KPA171202 (IB); 6, Δ*hyl* mutant (IB). See Table S3 for details on the HYL identification.

In order to confirm the production and secretion of HYL, strains KPA171202, ∆*hyl* mutant, and the type IA strains 266 and 12.1.L1 were grown in broth until stationary growth phase. Secreted proteins in the culture medium were harvested by precipitation and separated on a SDS-PAGE gel. Protein bands of the molecular weight between 80–90 kDa were excised and analyzed by mass spectrometry. HYL-IA and HYL-IB/II could both be identified as secreted proteins (Figure 4, Table S3). It appeared that HYL-IB/II in strain KPA171202 was stronger produced than HYL-IA in the strains 266 and 12.1.L1, although a clear quantitative comparison of HYL production levels between different strains was not possible due to a lack of a stably secreted reference protein.

### 3.3. A P. acnes Mutant that Lacks Hyl is Not Able to Degrade Hyaluronic Acid

**Figure 5:**
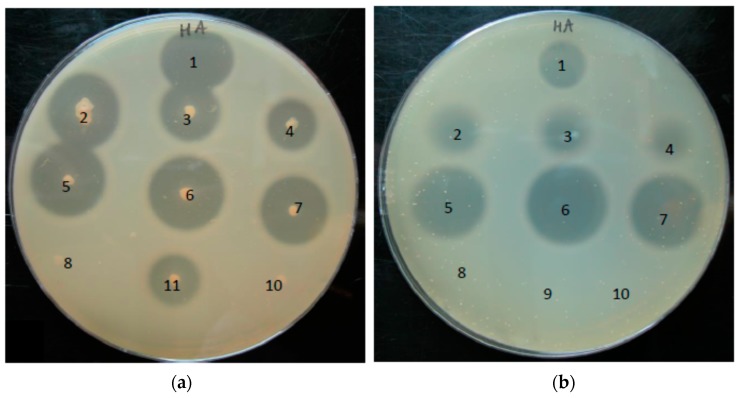
Detection of hyaluronate lyase activity of *P. acnes* strains with a HA plate assay. (**a**) Colonies of *P. acnes* were point-inoculated onto HA-containing plates and incubated for 24 h under anaerobic conditions. (**b**) 10 µL of supernatant harvested from *P. acnes* cultures grown until the stationary growth phase were spotted on HA-containing plates, and incubated for 24 h under anaerobic conditions. Plates were flushed with 2N acetic acid for 15 min for the detection of HA degradation. Numbers: 1, positive control (HYL from *Streptococcus pyogenes*); 2, strain 266 (IA); 3, strain 12.1.L1 (IA); 4, strain 15.1.R1 (IA); 5, strain KPA171202 (IB); 6, strain P6 (IB); 7, strain CCUG33950 (II); 8, strain Δ*hyl* mutant (IB); 9, negative control (BHI medium); 10, strain PMH5 (III); 11, strain 3.6.A1 (IA). The pictures are representative of three independent experiments.

In order to confirm that the HA-degrading activity is encoded by *hyl* we generated a *hyl* knockout mutant (∆*hyl*) in the wild-type strain KPA171202 (type IB). First, it was verified that the mutant does not possess the *hyl* gene (Figure S3). This was further confirmed on protein level: in contrast to the wild-type strain, no HYL protein could be detected in the supernatant of the ∆*hyl* mutant strain (Figure 4). Next, the mutant strain was tested for HYL activity in a HA-containing agar plate assay. The wild-type strain but not the mutant strain was able to produce clear zones in agar plates, indicative of HYL activity (Figure 5). Thus, this experiment confirms that *hyl* is responsible for *P. acnes*’ HA-degrading activity. Efforts to create a *hyl* knockout mutant in a type IA strain failed.

### 3.4. Phylotype-Specific Differences in Hyaluronate Lyase Activity

Next, we investigated if HYL-IB/II and HYL-IA differed in their HA-degrading activities. HYL activity was determined using HA-containing agar plates. Colonies of the investigated strains were inoculated onto the plates and incubated for 24 h. Both, phylotype IA strains and phylotype IB/II strains expressed HYL activity, while HYL activity was absent from the Δ*hyl* mutant strain and the phylotype III strain (Figure 5a). Interestingly, differences in the size of the clear zones around the colonies of type IA and type IB/II strains, respectively, were observed: HYL-IB/II-producing strains exhibited larger clear zones around colonies compared to HYL-IA-producing strains. In order to achieve a more quantitative comparison between different strains we applied supernatants of the *P. acnes* strains grown to the stationary growth phase in equal amounts to HA-containing agar plates (Figure 5b). This confirmed the presence of HYL activity in the culture supernatant and showed again that supernatants harvested from type IB/II strains produced larger clear zones in comparison to those obtained from phylotype IA strains. Taken together, the results of the HA plate assay revealed that both HYL-IA and HYL-IB/II activities could be detected, with a superior HA-degradation ability of HYL-IB/II.

A different assay to determine HA degradation activity, a HA turbidity assay, was also tested. This turbidimetric assay exploits the high turbidity of non-degraded HA, measured at OD_540nm_; decrease of turbidity due to degradation of HA can be quantified. Presence of HYL was investigated in the culture supernatants of *P. acnes* strains grown to the exponential growth phase (24 h), stationary growth phase (48 h, 72 h) and late-stationary growth phase (144 h). Corresponding growth curves of the tested strains were recorded to exclude an effect of growth differences when comparing HYL-IA and HYL-IB/II (Figure S5). The ∆*hyl* mutant and the PMH5 strains exhibited only very weak HA degradation activity (Figure S6). In contrast, the type IA and IB strains exhibited significant HA degradation activity, albeit, and in contrast to the HA plate assay, no differences between type IA and IB strains were observed.

### 3.5. Substrate Range of Hyaluronate Lyases of P. acnes

**Figure 6:**
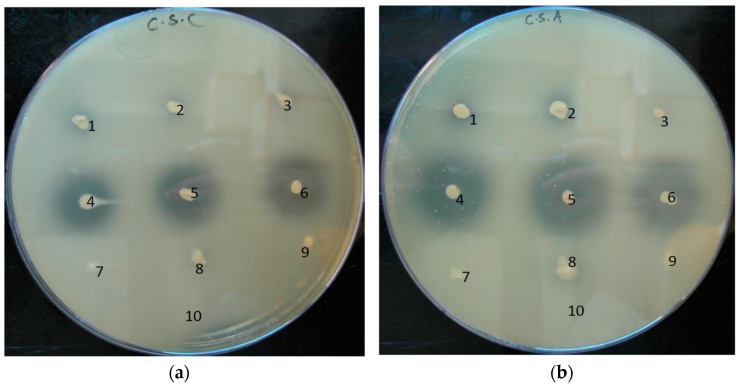
Chondroitin sulfate degradation by HYL activity of *P. acnes* strains. Colonies of *P. acnes* were point-inoculated onto CSC (**a**) and CSA (**b**)-containing plates and incubated under anaerobic conditions. CSA and CSC were degraded by HYL-IB/II, but only weakly by HYL-IA. Numbers: 1, strain 266 (IA); 2, strain 12.1.L1 (IA); 3, strain 15.1.R1 (IA); 4, strain KPA171202 (IB); 5, strain P6 (IB); 6, strain CCUG33950 (II); 7, ∆*hyl* mutant (IB); 8, strain 3.6.A1 (IA); 9, strain PMH5 (III); 10, control (BHI medium). The pictures are representative of three independent experiments.

The extracellular matrix of human skin is a complex network composed of a variety of GAGs, including HA and chondroitin sulfates. We wanted to investigate if *P. acnes* could degrade other GAGs. Chondroitin sulfate-containing plates were prepared containing chondroitin 4-sulfate (CSA), dermatan sulfate (CSB), and chondroitin 6-sulfate (CSC), and *P. acnes* colonies were point-inoculated onto the plates. The results of a semi-quantitative plate assay showed that none of the investigated strains could degrade CSB (Figure S7). However, some strains could degrade CSA and CSC, albeit forming smaller clear zones as compared to HA degradation after prolonged incubation (Figure 6). In analogy to the HA plate assay, the clear zones were particularly more pronounced for type IB/II strains compared to type IA strains; after short-term (24 h) incubation, clear zones were first only visible around colonies of type IB/II strains (Figure S7). The ∆*hyl* mutant strain did not exhibit any degradation of CSA, CSB and CSB. Thus, these findings show that HYL-IB/II of *P. acnes* can also degrade other GAGs, i.e., CSA and CSC, but with at a reduced rate compared to HA degradation. HYL-IA exhibits only very weak CSA/CSC-degrading activities.

### 3.6. Different Hyaluronic Acid Fragment Sizes Produced by P. acnes Hyaluronate Lyases

**Figure 7:**
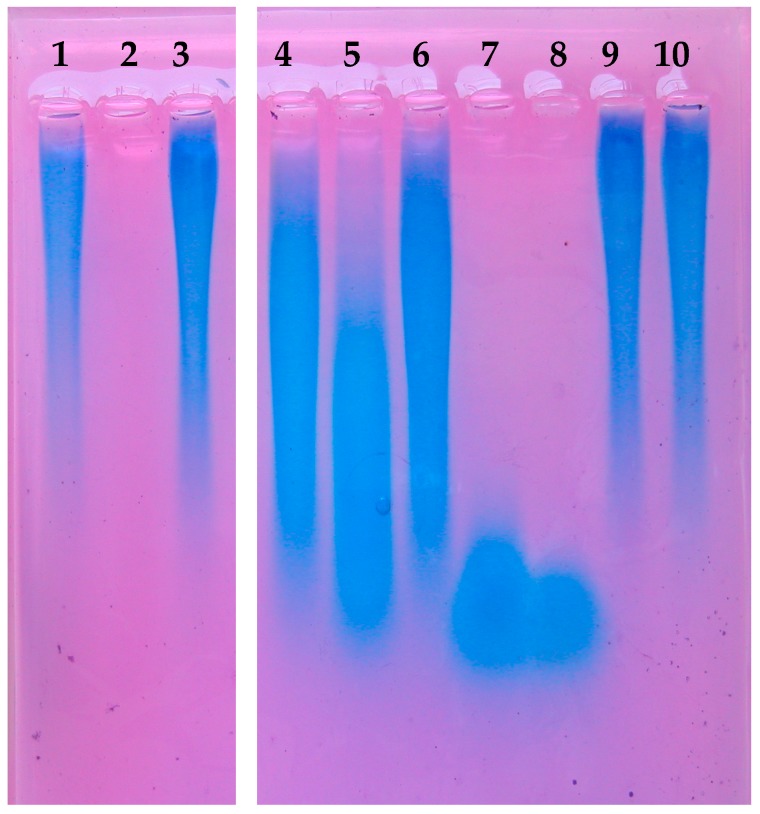
Hyaluronic acid fragments produced by the activity of HYLs of *P. acnes*. HA was incubated with controls and culture supernatants of *P. acnes* strains grown to the stationary phase. Visualization of HA fragments was done on a stained 1% agarose gel. HYL-IA produced larger HA fragments, whereas HYL-IB/II activity resulted in small HA fragments. Lanes: 1, HA input; 2, positive control (HYL from *S. pyogene*s); 3, culture medium (control); 4; strain 266 (IA); 5, strain 12.1.L1 (IA); 6, strain 15.1.R1 (IA); 7, strain KPA171202 (IB); 8, strain CCUG33950 (II); 9, strain PMH5 (III); 10, strain ∆*hyl* mutant. The picture is representative of three independent experiments.

The two HYL variants of *P. acnes*, HYL-IA and HYL-IB/II, harbor different properties with regard to their efficiency to degrade HA, CSA, and CSC. Next, we wanted to investigate if the variant difference affects the products, i.e., the produced HA fragments. This is biologically relevant since different HA fragment sizes, i.e., high molecular weight (HMW) and low molecular weight (LMW) HA fragments, have fundamentally different impacts on host cells and host tissues [52,53,54]. To test this, culture supernatants of the investigated *P. acnes* strains were incubated with 1% HA. The produced HA fragments were visualized on a 1% agarose gel. The results demonstrated that the ∆*hyl* mutant strain and PMH5 (type III) did not display any indication of HA degradation, as seen in comparison to native HA (Figure 7). HYL-IB/II-containing supernatants degraded HA into small homogeneous fragments. In contrast, HYL-IA degradation resulted in incomplete degradation of HA, resulting in HA fragments of various sizes. It was further examined if prolonged incubation with HYL-IA would eventually lead to further degradation of HA as observed for HYL-IB/II. However, extended incubation times did not resolve in complete degradation of HA by HYL-IA (Figure S8). Taken together, these results showed that the two HYL variants of *P. acnes* produced HA fragments of different sizes.

To further investigate the two HYL variants of *P. acnes* we tried to clone the corresponding genes and recombinantly express them in different yeast and bacterial systems. This turned out to be possible for the HYL-IA variant but not for HYL-IB/II, even with different expression systems and cloning conditions (data not shown). It appeared that the HYL-IB/II variant is toxic for an unknown reason.

## 4. Discussion

Our study showed that HYL is a widespread enzyme found in all phylogenetic types of *P. acnes* except for phylotype III, a type that is relatively rarely found on human skin of the face and upper back [20]. Furthermore, we showed that *hyl*-encoding *P. acnes* strains produce and secrete the HA-degrading enzyme. Thereby, two distinct variants of the enzyme could be differentiated, one variant present in *P. acnes* type IA strains, and the other one in type IB and type II strains. These two variants differ phenotypically, with HYL-IB/II having a superior ability to degrade HA into short fragments of uniform size, whereas HYL-IA activity produces larger HA fragments of various sizes.

*P. acnes*’ HYLs share homology to HYLs of other species such as *Streptococcus pyogenes* and *Streptomyces coelicolor* on amino acid sequence level [27,28,29,30,31,32]. HYLs from streptococci belong to the group I bacterial HYLs that degrade HA completely into disaccharide units, following a processive degradation pattern. This contrasts with HYLs of *Streptomyces* species, which belong to group II bacterial HYLs, that degrade HA by means of a non-processive degradation mechanism into a mixture of unsaturated oligosaccharides [27,28]. Genome context analyses in *P. acnes* suggest that the two variants HYL-IA and HYL-IB/II have been independently acquired in the evolutionary history of *P. acnes*; this was also recently proposed by Scholz et al. [21]. HYL-IA is encoded on a 6.3 kb genomic island specific to type IA strains, and HYL-IB/II is encoded on a 10 kb island specific to type IB and II strains. We speculate that the two HYL variants encoded on the phylotype-specific islands could have been independently acquired from two different sources: HYL-IA could potentially originate from *Streptomyces* (group II), as these HYLs degrade HA into various sizes of oligosaccharides. In contrast, HYL-IB/II could have been obtained from *Streptococcus* (group I), as these HYLs share similar HA degradation patterns. Further studies are necessary to investigate the evolutionary origin of *P. acnes*’ HYLs.

One phenotypical difference between HYL-IA and HYL-IB/II is the superior HA-degrading activity of HYL-IB/II as judged from the HA plate assay. Our study does not give a clear answer to the reasons of this difference, due to the impossibility to recombinantly produce HYL-IB/II, and subsequently compare recombinant HYL-IA and HYL-IB/II at equimolar amounts. One possibility is that the difference is due to the larger HYL amounts produced by phylotype IB/II strains, which is suggested by the secretome analysis performed here and previously [48]. Alternatively, HYL-IB/II might have a superior HA-degrading activity compared to HYL-IA, due to a higher substrate affinity and thus elevated substrate turnover. A previous study proposed a HA-binding site (RKVASSSTK); an antibody against this domain was inhibitory [38]. Interestingly, this binding motif is present only in the HYL-IB/II variant, but not in HYL-IA. This could indicate superior substrate-binding of HYL-IB/II. In this regard, it should be mentioned that a previous study failed to detect HA-degrading activity in type IA strains (in contrast to type IB and type II strains), as judged from a HA plate assay [7]. The result contradicts the data obtained in this study, albeit we detected a weaker HA-degrading activity of type IA strains. Possible explanations for this discrepancy are the use of a HA substrate from a different source or different culture and incubation conditions for the plate assay. A more recent study showed a widespread HA-degrading ability of the vast majority of *P. acnes* strains: Tyner & Patel detected the presence of *hyl* and HYL activity in 97% of the tested clinical isolates of *P. acnes* [39].

Regarding the substrate range of *P. acnes* HYLs, none of the investigated strains could degrade CSB. This result is supported by previous work on *P. acnes* HYL [37]. In analogy to HA, plate assays showed that HYL-IB/II had a superior ability to cleave CSA and CSC, whereas HYL-IA exhibited only a weak CSA/CSC-degrading ability. The limited ability of bacterial HYLs to degrade chondroitin sulfates seems to involve the sulfate groups of these GAGs, which have been shown to infer with the enzymatic activity [55].

The detected superior HA-, CSA- and CSC-degrading capacity of HYL-IB/II compared to HYL-IA could be related to colonization strategies in different ecological niches of the skin. Currently, there is incomplete knowledge about the *P. acnes* type distribution in different skin sites and skin tissue locations. Data analyzing skin swabs from different sites suggest that a mixture of different *P. acnes* types including IA, IB, IC, II, and III colonize human skin [15,20,56]. Facial skin and the skin of the upper body seems to be predominately colonized by type I strains, whereas proportions of type II and type III strains increase on the skin of the lower torso [20]. Acne vulgaris is associated with type IA strains, whereas type IB/II strains seem to be more often associated with soft and deep tissue colonization and infections [7,8,9,10,15]. Thus, HYL-IB/II might allow the efficient degradation of HA-/CSA-/CSC-containing extracellular matrix (ECM); this would allow tissue invasion and bacterial spread into deeper body sites. By contrast, the low HA-/CSA-/CSC-degrading activity of HYL-IA might not allow invasion.

The turbidimetric assay detected low HA-degrading activity in *hyl*-negative strains, i.e., the Δ*hyl* mutant and the type III strain. This indicates that other HA-degrading enzymes might be present in the genome of *P. acnes*. Genomic analysis revealed other putative enzymes with the possible ability to cleave HA. Putative β-exoglycosidases, including β-glucuronidase (PPA2333) and two β-*N*-acetylhexosaminidases (PPA2164, PPA1820) were found in the genome of KPA171202 [41]. However, no HA-degrading activity was detected in the plate assays for the Δ*hyl* mutant and the type III strains, suggesting that the putative β-glucuronidase and β-*N*-acetylhexosaminidases of *P. acnes* are not able to efficiently degrade HA and chondroitin sulfates, or are not functional in *P. acnes*.

The second difference of HYL-IA and HYL-IB/II activity concerned the produced HA fragments: HYL-IB/II can degrade HA into small homogenous fragments, whereas the less active HYL-IA produces larger oligosaccharides of various sizes. Even prolonged incubation did not resolve in full HA degradation by HYL-IA. This might be biological significant as the size of HA fragments determines the impact on host tissue. In its native state, HA in the ECM of the epidermis and dermis exists as a HMW molecule, where it is involved in normal cellular processes such as tissue integrity, scaffolding for cell migration, proliferation, and differentiation of cells [22,23,24]. Under certain conditions such as tissue invasion or injury, HA is fragmented to LMW fragments, i.e., oligosaccharides of various sizes. These LMW fragments serve as danger signals, and are involved in cell signaling events leading to the induction of inflammation, angiogenesis, and cancer-promoting processes [53,54,57,58,59]. The size of HA fragments defines which signaling events are initiated through the interaction with various cell types. We speculate that the various oligosaccharides produced by HYL-IA could be related to the inflammatory process induced by acne-associated type IA strains, as LMW HA fragments are able to stimulate or amplify the inflammatory response through interactions with cell surface receptors such as CD44 and TLR2 [34,60,61,62]. For instance, LMW HA fragments have previously been shown to induce the secretion of β-defensin 2 in human epidermal keratinocytes, as well as IL-6 and IL-8 in human dermal fibroblasts [63,64]. Thus, a pro-inflammatory response could be initiated, when phylotype IA strains (and their secreted proteins including HYL-IA) come in contact with the underlying dermis after comedome rupture. It has to be investigated in the future if HA fragments produced by HYL-IA could be involved in the induction or amplification of pro-inflammatory responses as seen in acne.

## 5. Conclusions

The present study sheds light on HYLs of different subtypes of *P. acnes*. It was shown that HYL is a widespread enzyme found in all clinical isolates of *P. acnes* except for phylotype III isolates, and that two distinct variants of HYL exist in the *P. acnes* population, that differ in their ability to degrade HA, CSA and CSC as well as produce distinct HA fragments. The study has some limitations, as it does not provide clear-cut evidence whether the difference in HYL activity is due to an actual difference in enzyme activity or a difference in the secreted levels of HYLs between type IA and type IB/II strains. Our study suggests that HYL is an important trait or ‘host adaptation factor’ for *P. acnes* that might play roles in nutrient acquisition, habitat colonization, and/or tissue invasion and bacterial spread. HYL-IB/II could be an important factor for the association of type IB and II strains with soft and deep tissue colonization and infection, as they harbor a highly active HYL with the ability to degrade major ECM components. The difference of the two *hyl* variants can also be used for diagnostic purposes to easily distinguish type IA, type IB/II and type III strains by one PCR.

## Supplementary Materials

The following are available online at www.mdpi.com/2076-2607/5/3/57/s1, Figure S1: Sequence alignment of HYL variants of the *P. acnes* phylotypes IA, IB, II, Figure S2: Extended genome context comparison of the region encoding HYL in *P. acnes*, Figure S3: PCR assay to differentiate the two HYL versions of *P. acnes*, Figure S4: Transcriptional start sites (TSS), putative -10 and -35 promoter regions and 5′-UTR of the *hyl* gene in *P. acnes*, Figure S5: Growth curves for investigated *P. acnes* strains, Figure S6: Turbidimetric assay to determine HA-degrading activity in *P. acnes*, Figure S7: Chondroitin sulfate (CS) degradation by HYL activity of *P. acnes* strains after 1 and 4 days of incubation, Figure S8: Hyaluronic acid fragments produced by the activity of HYLs of *P. acnes*, Table S1: Information about the strains used in this study, Table S2: Primers used in this study, Table S3: Identified HYL in the culture supernatant of *P. acnes* strains.
