# Supplementary material for: The Skin Bacterium Propionibacterium acnes Employs Two Variants of Hyaluronate Lyase with Distinct Properties
Source: Microorganisms. 2017 Sep 12;5(3):57. doi: 10.3390/microorganisms5030057 (PMC5620648; doi:10.3390/microorganisms5030057)
Supplement: Supplementary file 1 [file microorganisms-05-00057-s001.pdf]

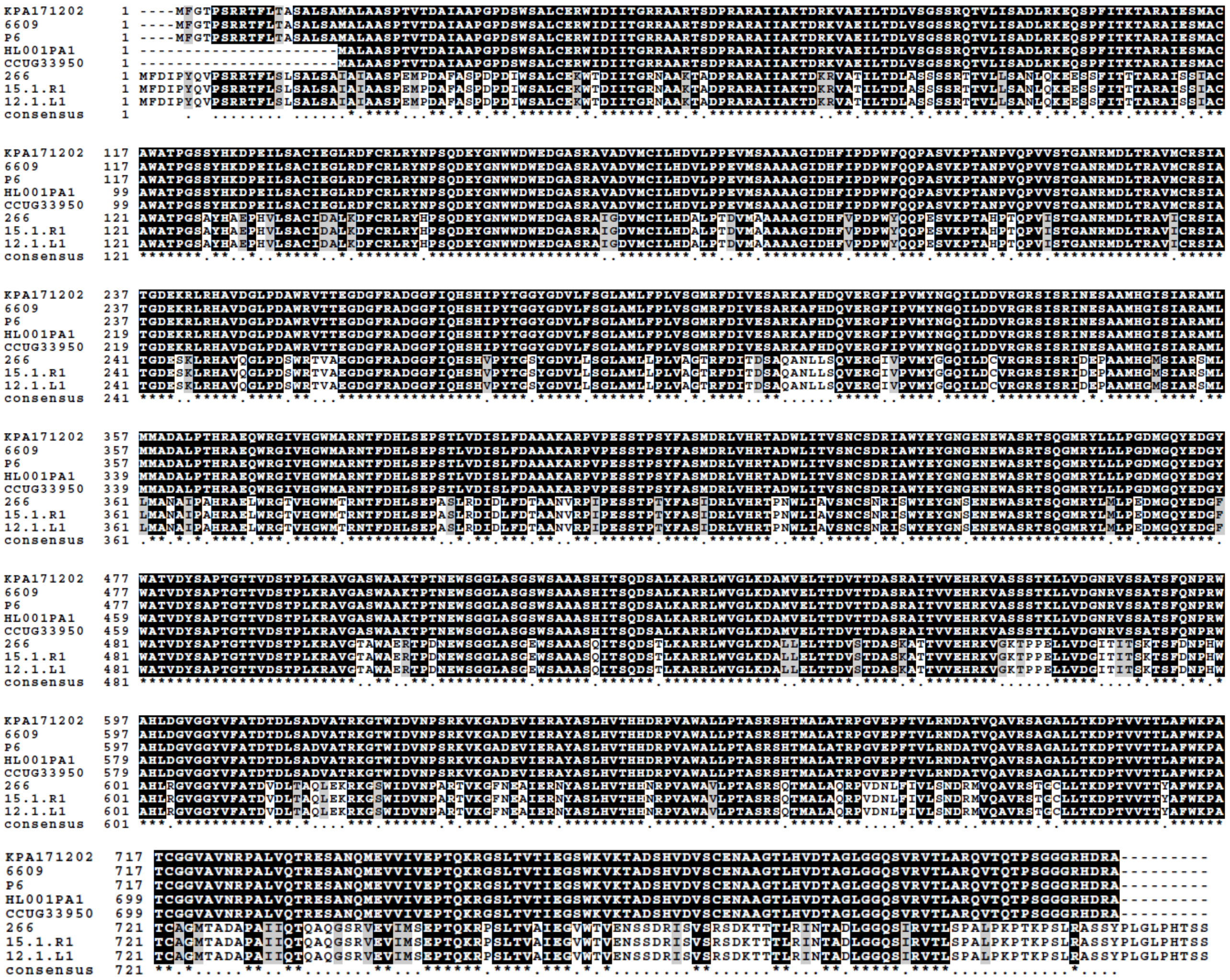

Figure S1. Sequence alignment of HYL variants of the *P. acnes* phylotypes IA, IB, II.

HYL sequences of the following strains were selected (in bracket their phylotypes): KPA171202 (IB); 6609 (IB); P6 (IB); HL001PA1 (II); CCUG33950 (II); 266 (IA); 15.1.R1 (IA); 12.1.L1 (IA).

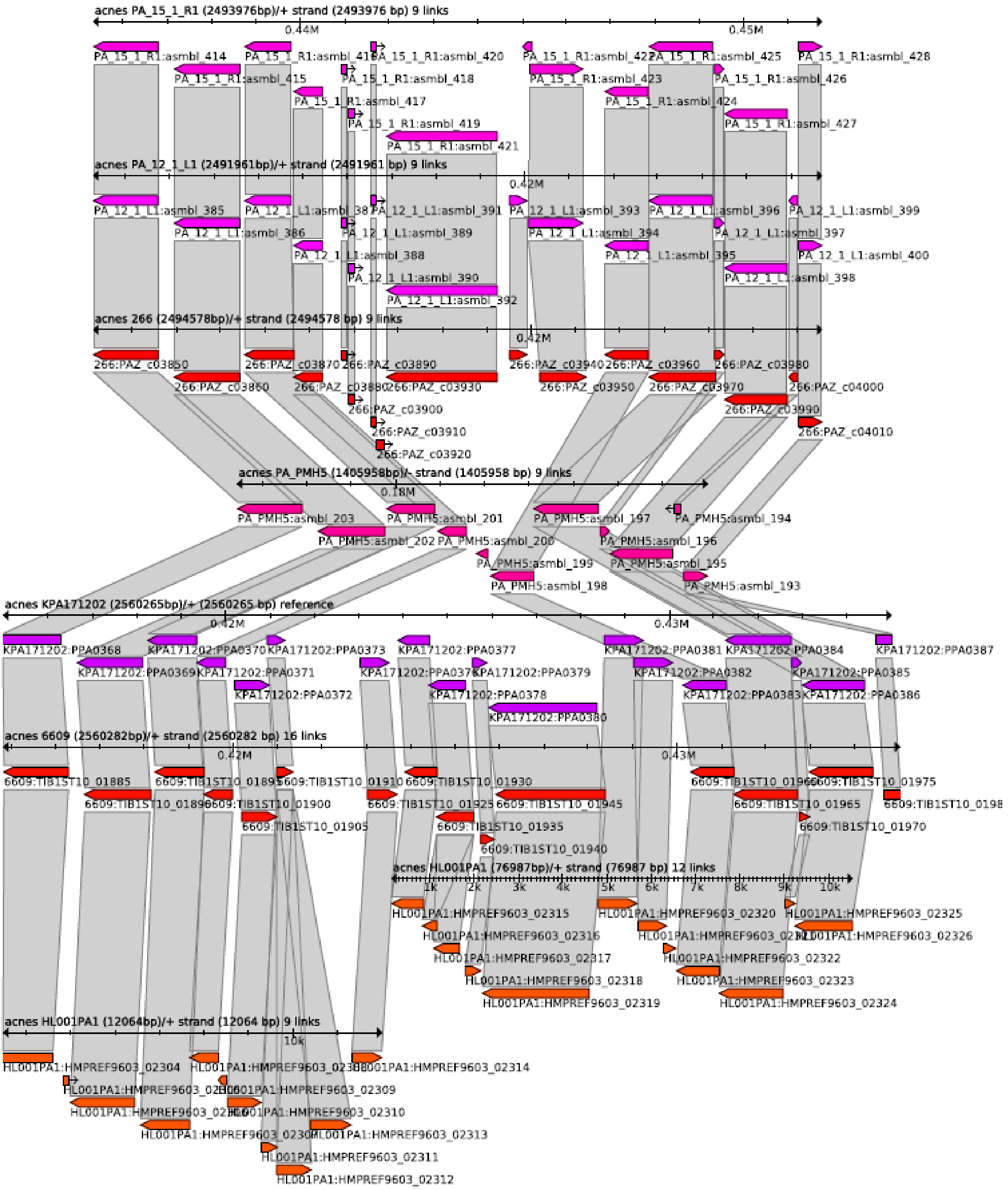

Figure S2. Extended genome context comparison of the region encoding HYL in *P. acnes*. The genomic region around *hyl* from strains 15.1.R1 (IA), 12.1.L1 (IA), 266 (IA), PMH5 (III), KPA171202 (IB), 6609 (IB), HL001PA1 (II) was compared using Sybil. The grey connecting blocks indicate amino acid sequences that share over 75% identity. Note that the genome of HL001PA1 is not closed.

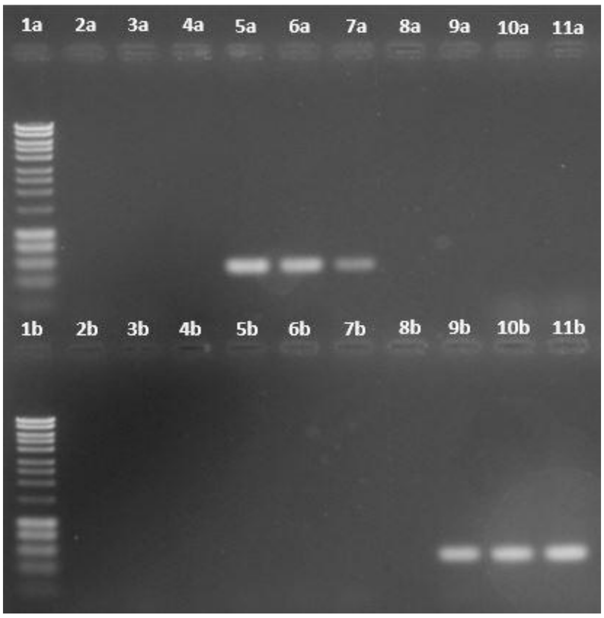

Figure S3. PCR assay to differentiate the two HYL versions of *P. acnes*. The detection of *hyl* in *P. acnes* strains was done by using HYL-IA- and HYL-IB/II-specific primers. PCR results with the primer pair for HYL-IA is shown in lanes 2a-11a, and with the primer pair for HYL-IB/II in lanes 2b-11b. Lanes: 1, DNA marker; 2, negative control (PCR water); ∆*hly* mutant; 4, empty; 5, 266 (IA); 6, 12.1.L1 (IA); 7, 15.1.R1 (IA); 8, PMH5 (III); 9, KPA171202 (IB); 10, P6 (IB); 11, CCUG33950 (II).

A

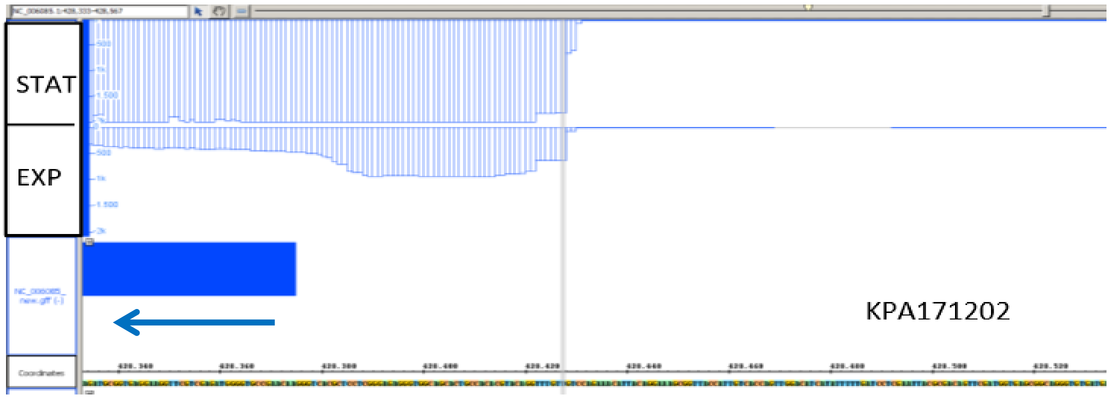

TSS

-35 -10

AAATATGATGTCCAACTGGTGACAATGGTAACCGCTTTCCTGTAATGTTTCTGGAC**A**ACAAA
CCTGTACGTGTGGCAGTGCTGCCACCCTCTCCCGAGGAGCGTGACCC**TTG**

B

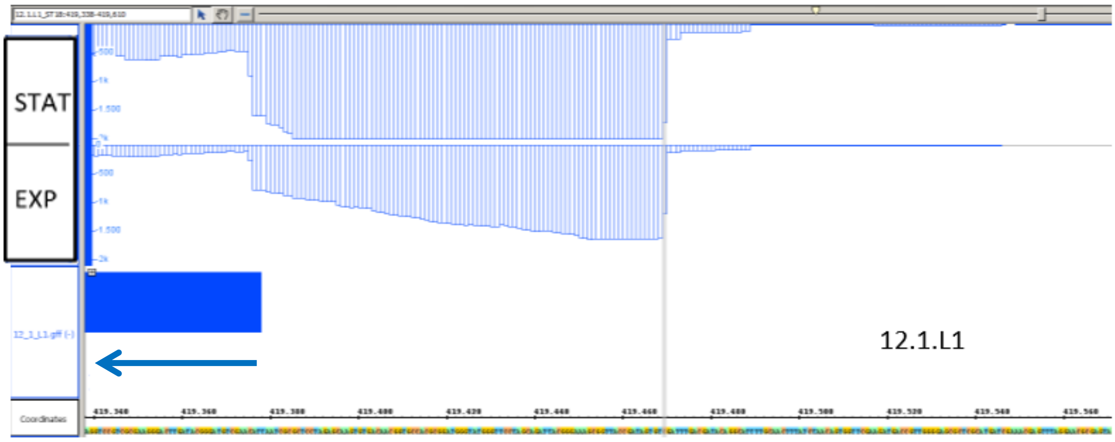

TSS

-35 -10

CCAACGGTCATCTTCGAACCACTGTTAGATAAAGTTGCAAAATGCCTGTATCGTCAAATC**G**ACACTAT
CGGTAACCGCTTTCCCGTAATCTGCTAGGAACCCATACCCATCCGCGTGGCACCGTTGTCACACTTGC
TCTAGGAGCGCGATTA**ATG**

Figure S4. Transcriptional start sites (TSS), putative -10 and -35 promoter regions and 5'-UTR of the *hly* gene in *P. acnes*. A: strain KPA171201 (IB); B: strain 12.1.L1 (IA).

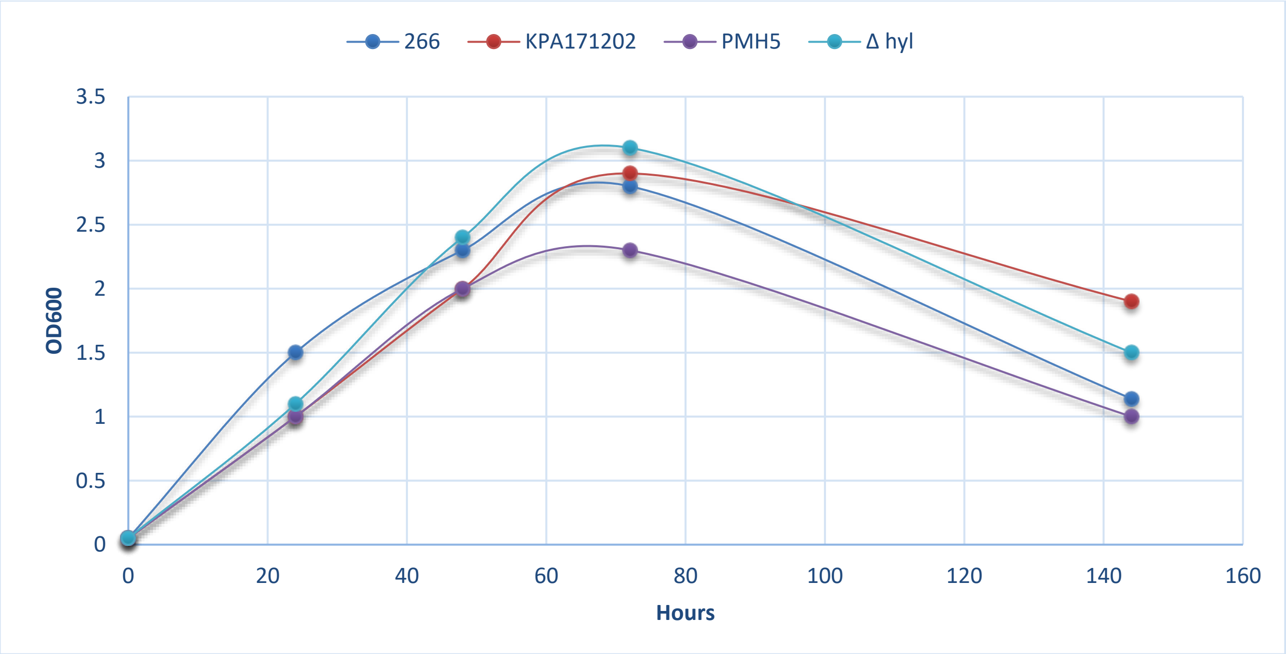

Figure S5. Growth curves for investigated *P. acnes* strains.
Strains were grown in BHI medium under anaerobic conditions at 37°C. OD was measured photometrically at 600 nm.

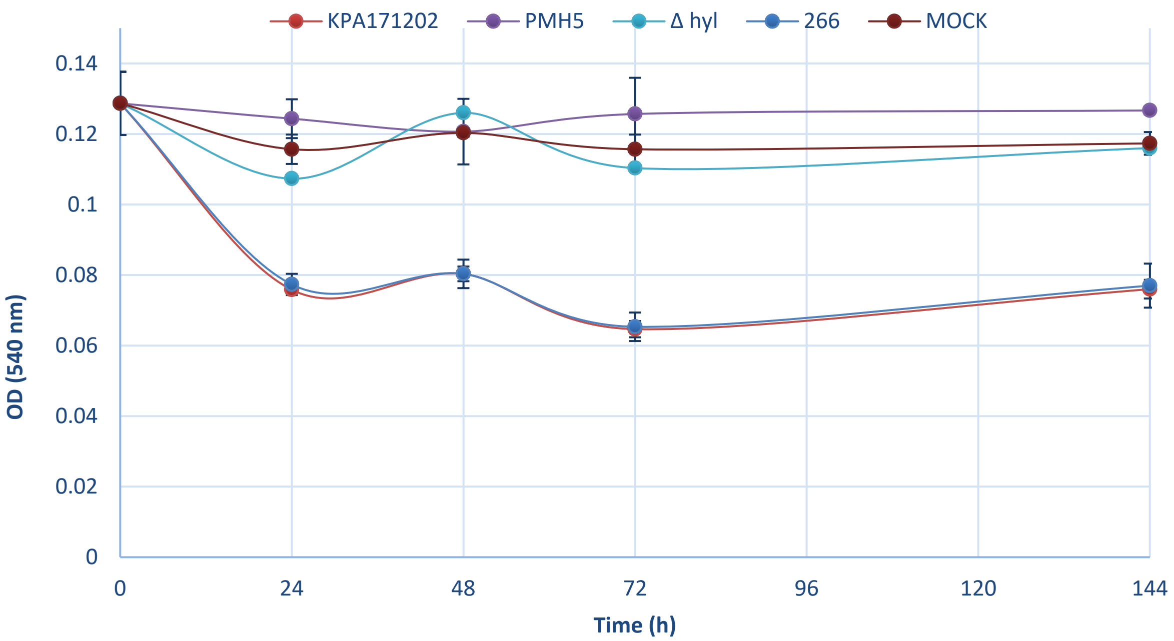

Figure S6. Turbidimetric assay to determine HA-degrading activity in *P. acnes*. Supernatants from strains 266, KPA171202, PMH5 and the Δ*hly* mutant were harvested from cultures grown to different time points (24 h, 48 h, 72 h, 144 h) and incubated with HA for 10 min. The turbidity of HA was determined after reaction with acidic albumin reagent at $OD_{540\ nm}$. The experiment was repeated twice, and each sample was done in triplicates. Bars represent the mean SD.

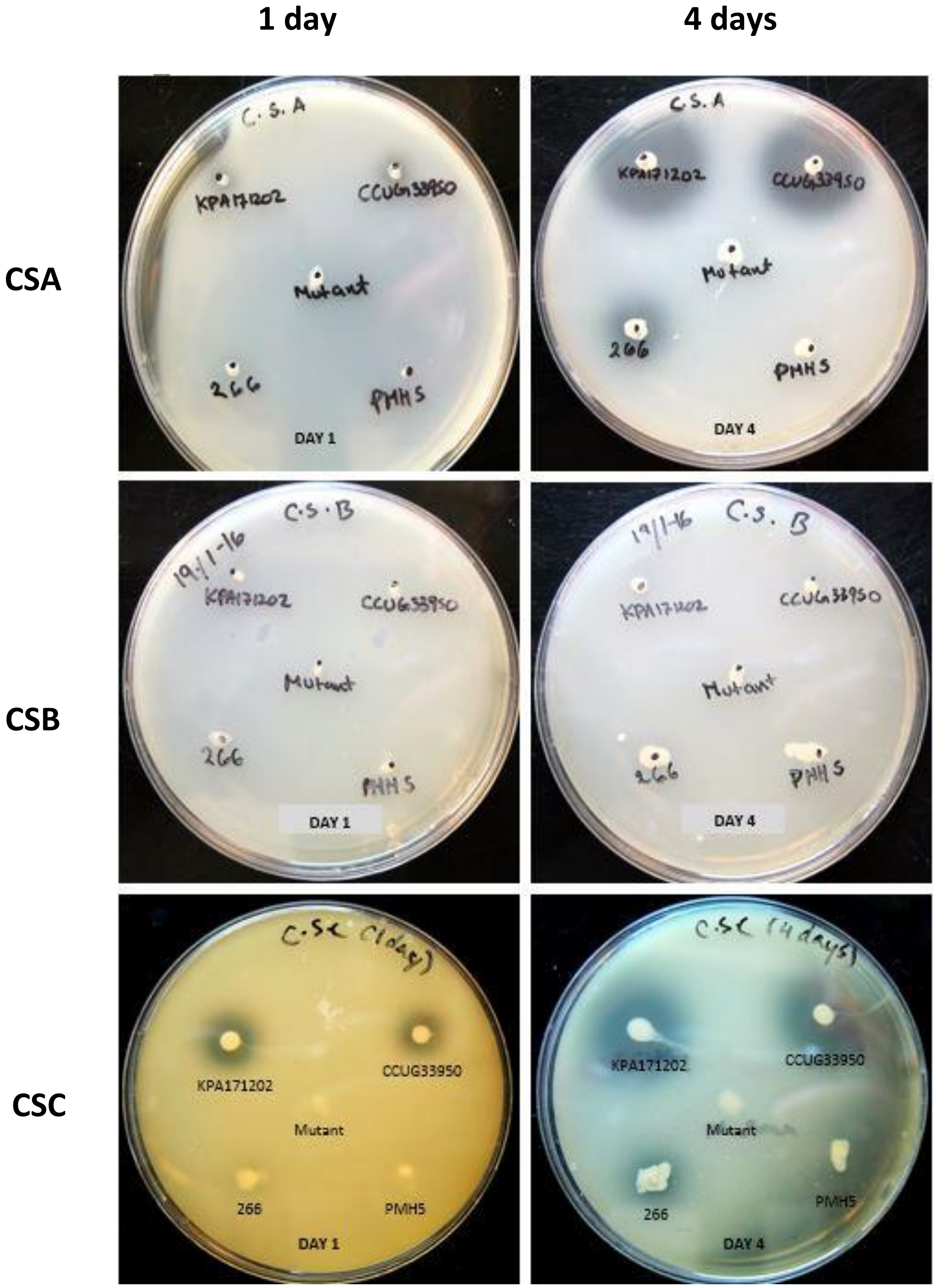

Figure S7. Chondroitin sulfate (CS) degradation by HYL activity of *P. acnes* strains after 1 and 4 days of incubation. Colonies of *P. acnes* strains KPA171202 (IB, upper left), CCUG33950 (II, upper right), 266 (IA, lower left), PMH5 (III, lower right) and the Δ*hyl* mutant (center) were point-inoculated onto CS-containing plates and incubated for 1 and 4 days. Plates were flushed with 2N acetic acid for 15 min to detect CS-degrading activity. None of the investigated strains showed any CSB (dermatan sulfate)-degrading activity, but type IB/II strains were able to degrade CSA and CSC. The type IA strain showed only weak CSA and CSC degradation after 4 days incubation.

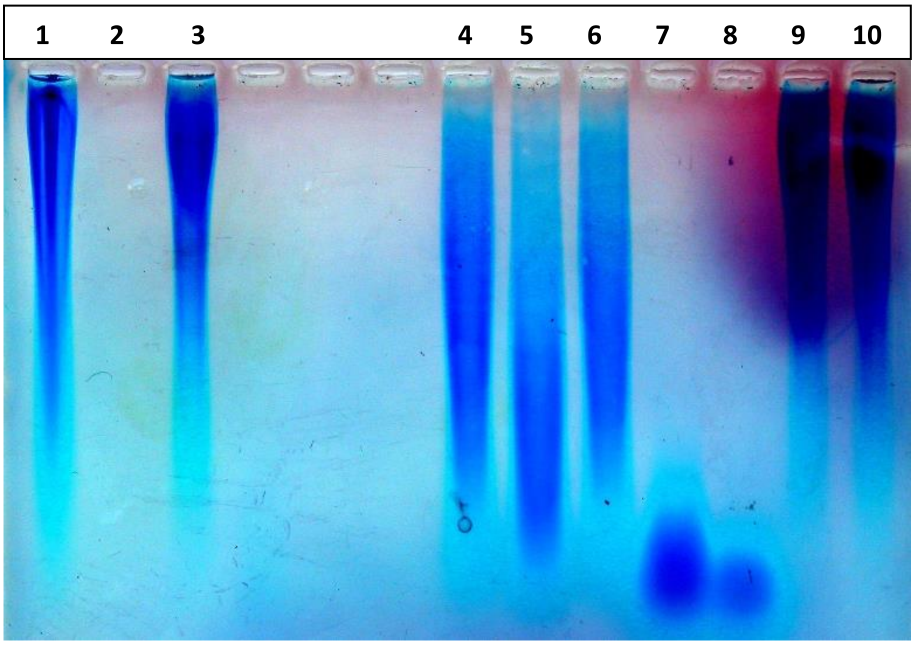

Figure S8. Hyaluronic acid fragments produced by the activity of HYLs of *P. acnes*.
HA was incubated with culture supernatants of *P. acnes* strains grown to the stationary phase for extended times (30 min instead of 10 min). Visualization of HA fragments on a stained 1% agarose gel. Extended incubation time did not resolve in complete degradation of HA by HYL-IA. Lanes: 1, HA input; 2, positive control (HYL from *S. pyogene*s); 3, culture medium (control); 4; strain 266 (IA); 5, strain 12.1.L1 (IA); 6, strain 15.1.R1 (IA); 7, strain KPA171202 (IB); 8, strain CCUG33950 (II); 9, strain PMH5 (III); 10, strain Δ*hly* mutant.

Table S1. Information about the strains used in this study.

Strain	Origin/reference	Phylotype	MLST/SLST *	*hyl* gene
**KPA171202**	skin/culture contaminant [41]	IB	CC36/H2	+ (HYL-IB/II)
**Δ*hyl* (KPA171202)**	this study	IB	CC36/H2	-
**P6**	prostatic tissue [66]	IB	CC36/H2	+ (HYL-IB/II)
**CCUG33950**	cerebrospinal fluid (CCUG strain collection) [7]	II	CC53/K5	+ (HYL-IB/II)
**12.1.L1**	skin/acne lesion [7,21]	IA	CC18/A1	+ (HYL-IA)
**15.1.R1**	skin/acne lesion [67]	IA	CC3/C1	+ (HYL-IA)
**266**	pleuropulmonary infection [48]	IA	CC18/A1	+ (HYL-IA)
**3.6.A1**	skin/healthy [7,21,67]	IA	singleton/E3	+ (HYL-IA)
**PMH5**	skin/progressive macular hypomelanosis lesion [68]	III	singleton/L1	-

* MLST/SLST schemes: references [7,11]

Additional references:

66. Fassi Fehri, L.; Mak, T.N.; Laube, B.; Brinkmann, V.; Ogilvie, L.A.; Mollenkopf, H.; Lein, M.; Schmidt, T.; Meyer, T.F.; Brüggemann, H. Prevalence of *Propionibacterium acnes* in diseased prostates and its inflammatory and transforming activity on prostate epithelial cells. *Int. J. Med. Microbiol*. **2011**, *301*, 69-78.

67. Brüggemann, H.; Lomholt, H.B.; Tettelin, H.; Kilian, M. CRISPR/cas loci of type II *Propionibacterium acnes* confer immunity against acquisition of mobile elements present in type I *P. acnes*. *PLoS One* **2012**, *7*, e34171.

68. Petersen, R.; Lomholt, H.B.; Scholz, C.F.; Brüggemann, H. Draft genome sequences of two *Propionibacterium acnes* strains isolated from progressive macular hypomelanosis lesions of human skin. *Genome Announc*. **2015**, *3*, pii: e01250-15.

Table S2. Primers used in this study.

Purpose	Primer sequence 5’-3’	
**Amplification of *hyl* of types IB/II**	HYL_IB/II_for<br>HYL_IB/II_rev	CTGCCGACTTCGGTACAACC<br>TTACGAGCCGATTCGACGAT
**Amplification of *hyl* of type IA**	HYL_IA_for<br>HYL_IA_rev	CGCCATCAAGGCACCAACAA<br>GTGATGGTAATGCCGTCGACTA
**Amplification of *hyl* upstream region**	PPA0380_1<br>PPA0380_2	CTCCTGGGTCCGCTGAGAGA<br>GCGGTACCGGGTCACGCTCCTCGGGAGA
**Amplification of *hyl* downstream region**	PPA0380_3<br>PPA0380_4	GCGGTACCTGTGCCGCCTCACCTAACTA<br>GCAGTGGGCTTCTCGGTGTA
**Amplification of erythromycin resistance gene**	ermE_for<br>ermE_rev	GAGCGCACCGACCCGGTCGT<br>TGCTGCGCCAGCGTTGTGCG

Table S1: Identified HYL in the culture supernatant of *P. acnes* strains.

Highlighted are the identified peptides (in red). Note that HYL-IA was the best match for strains 266 and 12.1.L1 and HYL-IB/II for strain KPA171202

Strain	Protein Name	Mass kDa	Sequence	Score	Matches	Sequences	Coverage %	Unique Peptides
**strain 266 (IA)**	**Hyaluronate lyase (HYL-IA)**	89,998	MFDIPYQVPSRRTFLSLSALSAIAIAASPEMPDAFASPDPDIWSALCEK**WTDIITGR**NAAKTADPRARAIIAKT<br>DKR**VATILTDLASSSSR**TTVLLSANLQK**EESSFITTTAR**AISSIACAWATPGSAYHAEPHVLSVCIDALKDFCRL<br>RYHPSQDEYGNWWDWEDGASRAIGDVMCILHDALPTDVMAAAAAGIDHFVPDPWYQQPESVKPTAHP<br>TQPVISTGANRMDLTRAVICRSIATGDESKLRHAVQGLPDSWR**TVAEGDGFR**ADGGFIQHSHVPYTGSYGD<br>VLLSGLAMLLPLVAGTRFDITDSAQANLLSQVERGIVPVMYGGQILDCVRGRSISR**IDEPAAMHGMSIARSM**<br>**LLMANAIPAHR**AELWR**GTVHGWMTRNTFDHLSEPASLR**DIDLFDTAANVRPIPESSTPTYFASIDRLVHRT<br>PNWLIAVSNCSNRISWYEYGNSENEWASRTSQGMRYLMLPEDMGQYEDGFWATVDYSAPTGTTVDSTPL<br>KR**AVGTAWAER**TPDNEWSGGLASGEWSAAASQITSQDSTLKARR**LWVGLK**DALLELTTDVSTDASKATTV<br>VEHRKVGK**TPPELLVDGITITSKTSFDNPHWAHLR**GVGGYVFATDVDLTAQLEKR**KGSWIDVNPAR**TVK**GF**<br>**NEAIER**NYASLHVTHHNRPVAWAVLPTASRSQTMALAQRPVDNLFIVLSNDR**MVQAVR**STGCLLTKAPTV<br>VTTYAFWKPATCAGMTADAPAIIQTQAQGSR**VEVIMSEPTQK**RPSLTVAIEGVWTVENSSDRISVSRSDKTT<br>TLR**INTADLGGQSIRVTLSPALPKPTKPSLR**ASSYPLGLPHTSS	3397	85	19	23,8	13
	**Hyaluronate lyase (HYL-IB/II)**	88,13	MFGTPSRRTFLTASALSAMALAASPTVTDAIAAPGPDSWSALCERWIDIITGRRAARTSDPRARAIIAKTDRK<br>VAEILTDLVSGSSRQTVLISADLRK**EQSPFITK**TARAIESMACAWATPGSSYHKDPEILSACIEGLRDFCRLRYN<br>PSQDEYGNWWDWEDGASRAVADVMCILHDVLPPEVMSAAAAGIDHFIPDPWFQQPASVKPTANPVQP<br>VVSTGANRMDLTRAVMCRSIATGDEKRLRHAVDGLPDAWR**VTTEGDGFR**ADGGFIQHSHIPYTGGYGDVL<br>FSGLAMLFPLVSGMR**FDIVESAR**KAFHDQVERGFIPVMYNGQILDDVRGRSISRINESAAMHGISIARAML<br>MMADALPTHRAEQWRGIVHGWMARNTFDHLSEPSTLVDISLFDAAAKARPVPESSTPSYFASMDRLVHRT<br>ADWLITVSNCSDRIAWYEYGNGENEWASRTSQGMRYLLLPGDMGQYEDGYWATVDYSAPTGTTVDSTPL<br>KRAVGASWAAKTPTNEWSGGLASGSWSAAASHITSQDSALKARR**LWVGLK**DAMVELTTDVTTDASRAITV<br>VEHRKVASSSTK**LLVDGNRVSSATSFQNPR**WAHLDGVGGYVFATDTDLSADVATRKGTWIDVNPSRKVKG<br>ADEVIERAYASLHVTHHDRPVAWALLPTASRSHTMALATRPGVEPFTVLR**NDATVQAVRSAGALLTK**DPTV<br>VTTLAFWKPATCGGVAVNRPALVQTRESANQMEVVIVEPTQKRGSLTVTIEGSWKVKTADSHVDVSCENA<br>AGTLHVDTAGLGGQSVRVTLARQVTQTPSGGGRHDRA	374	13	8	8,1	7

**strain 12.1.L1 (IA)**	**Hyaluronate lyase (HYL-IA)**	89,998	MFDIPYQVPSRRTFLSLSALSAIAIAASPEMPDAFASPDPDIWSALCEK**WTDIITGR**NAAKTADPRARAIIAKT<br>DKR**VATILTDLASSSSR**TTVLLSANLQK**EESSFITTTAR**AISSIACAWATPGSAYHAEPHVLSVCIDALKDFCRL<br>RYHPSQDEYGNWWDWEDGASRAIGDVMCILHDALPTDVMAAAAAGIDHFVPDPWYQQPESVKPTAHP<br>TQPVISTGANRMDLTRAVICR**SIATGDESKLR**HAVQGLPDSWR**TVAEGDGFR**ADGGFIQHSHVPYTGSYG<br>DVLLSGLAMLLPLVAGTRFDITDSAQANLLSQVERGIVPVMYGGQILDCVRGRSISR**IDEPAAMHGMSIARS**<br>**MLLMANAIPAHR**AELWR**GTVHGWMTRNTFDHLSEPASLR**DIDLFDTAANVRPIPESSTPTYFASIDRLVHR<br>TPNWLIAVSNCSNRISWYEYGNSENEWASRTSQGMRYLMLPEDMGQYEDGFWATVDYSAPTGTTVDSTP<br>LKR**AVGTAWAER**TPDNEWSGGLASGEWSAAASQITSQDSTLKARR**LWVGLK**DALLELTTDVSTDASKATT<br>VVEHRKVGK**TPPELLVDGITITSKTSFDNPHWAHLR**GVGGYVFATDVDLTAQLEKR**KGSWIDVNPAR**TVK<br>**GFNEAIER**NYASLHVTHHNRPVAWAVLPTASRSQTMALAQRPVDNLFIVLSNDR**MVQAVR**STGCLLTKAP<br>TVVTTYAFWKPATCAGMTADAPAIIQTQAQGSR**VEVIMSEPTQK**RPSLTVAIEGVWTVENSSDRISVSRSD<br>KTTTLR**INTADLGGQSIRVTLSPALPKPTKPSLRASSYPLGLPHTSS**	3446	86	21	26,8	15
	**Hyaluronate lyase (HYL-IB/II)**	88,13	MFGTPSRRTFLTASALSAMALAASPTVTDAIAAPGPDSWSALCERWIDIITGRRAARTSDPRARAIIAKTDRK<br>VAEILTDLVSGSSRQTVLISADLRK**EQSPFITK**TARAIESMACAWATPGSSYHKDPEILSACIEGLRDFCRLRYN<br>PSQDEYGNWWDWEDGASRAVADVMCILHDVLPPEVMSAAAAGIDHFIPDPWFQQPASVKPTANPVQP<br>VVSTGANRMDLTRAVMCRSIATGDEKRLRHAVDGLPDAWR**VTTEGDGFR**ADGGFIQHSHIPYTGGYGDVL<br>FSGLAMLFPLVSGMR**FDIVESARKAFHDQVER**GFIPVMYNGQILDDVRGRSISR**INESAAMHGISIARAML**<br>**MMADALPTHR**AEQWRGIVHGWMARNTFDHLSEPSTLVDISLFDAAAK**ARPVPESSTPSYFASMDR**LVHR<br>TADWLITVSNCSDRIAWYEYGNGENEWASRTSQGMRYLLLPGDMGQYEDGYWATVDYSAPTGTTVDSTP<br>LKR**AVGASWAAK**TPTNEWSGGLASGSWSAAASHITSQDSALKARR**LWVGLK**DAMVELTTDVTTDASR**AI**<br>**TVVEHR**KVASSSTK**LLVDGNRVSSATSFQNPR**WAHLDGVGGYVFATDTDLSADVATRKGTWIDVNPSRKV<br>K**GADEVIER**AYASLHVTHHDRPVAWALLPTASRSHTMALATRPGVEPFTVLR**NDATVQAVRSAGALLTK**D<br>PTVVTTLAFWKPATCGGVAVNRPALVQTR**ESANQMEVVIVEPTQKR**GSLTVTIEGSWKVKTADSHVDVSC<br>ENAAGTLHVDTAGLGGQSVRVTLAR**QVTQTPSGGGR**HDRA	2343	70	19	21,3	18
**strain KPA (IB)**	**Hyaluronate lyase (HYL-IB/II)**	88,13	MFGTPSRRTFLTASALSAMALAASPTVTDAIAAPGPDSWSALCER**WIDIITGR**RAARTSDPRARAIIAKTDR**K**<br>**VAEILTDLVSGSSRQTVLISADLRKEQSPFITK**TARAIESMACAWATPGSSYHKDPEILSACIEGLRDFCRLRYN<br>PSQDEYGNWWDWEDGASRAVADVMCILHDVLPPEVMSAAAAGIDHFIPDPWFQQPASVKPTANPVQP<br>VVSTGANRMDLTRAVMCRSIATGDEKRLR**HAVDGLPDAWRVTTEGDGFR**ADGGFIQHSHIPYTGGYGDV<br>LFSGLAMLFPLVSGMR**FDIVESARKAFHDQVER**GFIPVMYNGQILDDVRGRSISR**INESAAMHGISIARAM**<br>**LMMADALPTHR**AEQWR**GIVHGWMAR**NTFDHLSEPSTLVDISLFDAAAK**ARPVPESSTPSYFASMDR**LVH<br>RTADWLITVSNCSDRIAWYEYGNGENEWASRTSQGMRYLLLPGDMGQYEDGYWATVDYSAPTGTTVDST<br>PLKR**AVGASWAAK**TPTNEWSGGLASGSWSAAASHITSQDSALKARR**LWVGLK**DAMVELTTDVTTDASR**A**<br>**ITVVEHRK**VASSSTK**LLVDGNRVSSATSFQNPR**WAHLDGVGGYVFATDTDLSADVATR**KGTWIDVNPSRK**<br>**VKGADEVIER**AYASLHVTHHDRPVAWALLPTASR**SHTMALATRPGVEPFTVLRNDATVQAVRSAGALLTK**<br>DPTVVTTLAFWKPATCGGVAVNRPALVQTR**ESANQMEVVIVEPTQKR**GSLTVTIEGSWKVKTADSHVDVS<br>CENAAGTLHVDTAGLGGQSVRVTLAR**QVTQTPSGGGR**HDRA	6017	171	34	32,1	34
